# Behavioral Responses of *Chironomus aprilinus* Larvae as Proxies for Cyanobacterial Metabolite Interactions: Insights from Ternary Combinations

**DOI:** 10.1021/acs.est.4c07823

**Published:** 2024-10-15

**Authors:** Adam Bownik, Donald Wlodkowic, Barbara Pawlik-Skowrońska, Tomasz Mieczan

**Affiliations:** † Department of Hydrobiology and Protection of Ecosystems, University of Life Sciences in Lublin, Dobrzańskiego 37, 20-262 Lublin, Poland; ‡ The Neurotox Lab, School of Science, RMIT University, Plenty Road, P.O. Box 71, Bundoora, VIC 3083, Australia

**Keywords:** cyanobacterial products, ecotoxicity, aquatic invertebrates, movement speed

## Abstract

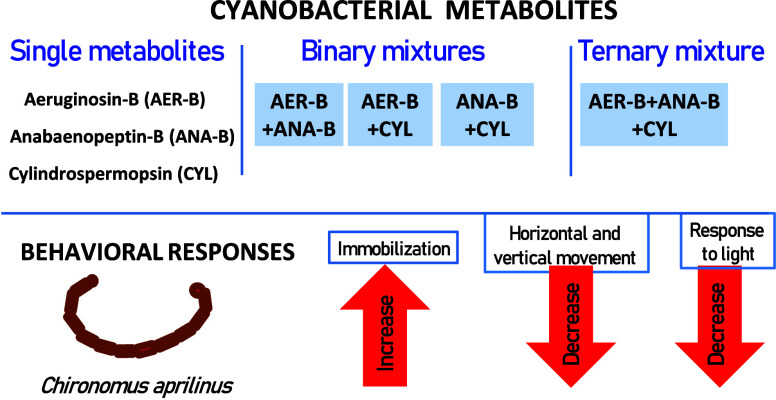

This study aimed to assess the behavioral responses (immobilization, horizontal and vertical motility, and response to light) of *Chironomus aprilinus* larvae exposed to individual cyanobacterial metabolites aeruginosin 98B (AER-B), anabaenopeptin-B (ANA-B), and cylindrospermopsin (CYL), and their binary and ternary mixtures. The investigation revealed that single metabolites ANA-B and CYL exhibited the highest potency in immobilizing the larvae. Notably, the binary mixture AER-B+CYL induced a remarkably strong synergistic interaction, while other tested binary and ternary mixtures demonstrated antagonistic effects. Both individual metabolites and their mixtures led to a decrease in larval movement speed, with the AER-B+CYL combination showing a very synergistic effect, and strong antagonistic interactions between the oligopeptides in the ternary mixture. Conversely, while AER-B and the binary mixture ANA-B+CYL stimulated vertical movement, other single metabolites and binary and ternary mixtures decreased this parameter. Antagonistic interactions were observed in all mixtures. ANA-B emerged as the most potent inhibitor, yet all tested metabolites and their mixtures decreased larval response to light, displaying synergistic interactions, except for the AER-B+ANA-B mixture at 250 μg L^–1^ + 250 μg L^–1^. These findings underscore the sensitivity of *Chironomus* larvae behavioral parameters as indicators of environmental stressors and mixtures. Consequently, they are recommended for assessing toxic effects induced by cyanobacterial products and other bioactive chemicals.

## Abstract 2

Little is known about the effects induced by the mixtures of cyanobacterial metabolites on the aquatic invertebrates. We propose novel behavioral responses of *Chironomus aprilinus* larvae as indicators of their toxicity.

## 1 Introduction

Cyanobacteria, as phototrophic microorganisms, produce a diverse array of metabolites with significant biological activity. Several studies have demonstrated the hepatotoxic, neurotoxic, or dermal toxicity of these metabolites in various organisms, including mammals, fish, aquatic or terrestrial invertebrates, and humans.^1−3^ The chemical diversity of cyanobacterial products encompasses oligopeptides, alkaloids, and lipopolysaccharides.^1,2^ Although effects induced by major cyanobacterial cyanotoxins such as microcystins and anatoxin-a are well documented, little is known about the broader category of these metabolites, frequently detected in water reservoirs worldwide.^4^

Aeruginosins (AERs), for instance, are cyanobacterial linear tetrapeptides synthesized by various genera such as *Microcystis*, *Nostoc, Nodularia*, and *Planktothrix*, though their study remains limited.^5−7^ These peptides, identified as aminopeptidase inhibitors,^7,8^ are rarely investigated cyanobacterial oligopeptides, like the other microginin-FR1, inducing various toxic effects such as behavioral disturbances and cytotoxicity in rotifers.^9^ AERs have also been shown to exert toxicity manifested by decreased survival rates or to accumulate in tissues of aquatic invertebrates such as *Thamnocephalus platyurus* and *Artemia franciscana*.^7,10^ The knowledge on the environmental concentrations of aeruginosins is very scarce; nevertheless, AER 583 was detected at 0.2 ± 0.3 μg L^–1^ in Lake Steinsfjorden.^11^

Cyclic hexapeptides known as anabaenopeptins (ANAs) have been detected in various cyanobacterial genera, including *Planktothrix, Microcystis, Nostoc*, and *Nodularia.*(12,13) ANAs exhibit a wide concentration range in water bodies, spanning 3–1000 μg L^–1^, surpassing levels reached by microcystins.^14^ ANAs have been found to induce behavioral disturbances in aquatic invertebrates such as nematodes,^15^ cladocerans,^16^ and rotifers.^9^

Cylindrospermopsin (CYL), an alkaloid initially noted in *Cylindrospermopsis raciborskii*, has also been found in other genera such as *Chrysosporum ovalisporum* and *Synechococcus* sp.^17,18^ While CYL’s dissolved concentration in water reservoirs averages 1.7 μg L^–1^, its metabolite 7-deoxy-cylindrospermopsin (also produced by cyanobacterial species *Raphidiopsis mediterranea* Skuja) may reach concentrations of up to 1065 μg L^–1^.^19,20^ This cyanotoxin was also detected in the benthic zone at a range of 0.00196–1580 μg L^–1^.^21,22^ CYL has been implicated in toxic effects on both plants (decreased growth rate, disturbances in oxygen production, and pigment content changes)^23^ and animals, such as cytotoxic and behavioral changes in invertebrates,^9^ lethality, hepatotoxicity, dermatotoxicity, genotoxicity, oxidative stress, and neurotoxicity in lower and higher vertebrates.^24,25^

*Chironomus*, belonging to the *Diptera* order and *Chironomidae* family, plays diverse ecological roles, serving as a crucial food source for fish, shoreline birds, and predatory insects. The larvae, in particular, spend their developmental stages in aquatic environments, consuming detritus and thus playing a substantial role in recycling organic debris.^26^ The behavioral characteristics of both adult and larval *Chironomidae*, such as attraction to light (negative phototaxis) and reactions to gravity (positive georesponses), have been studied.^27,28^*Chironomus* larvae have also been proposed as model invertebrates in ecotoxicological studies, emphasizing their specific behavioral responses as valuable indicators.^29−33^

Despite the documented effects of various cyanobacterial strains on *Chironomus* larvae, particularly *Trichormus variabilis* and *Anabaena*, such as increased mortality, decreased larval mass, oxidative stress, DNA damage, and reduction of hemoglobin concentration,^32,33^ little information exists regarding the impact of individual cyanobacterial metabolites, such as ANAs, AERs, and CYL on the behavioral responses of *Chironomus*. Furthermore, there is a knowledge gap regarding the interactive effects of these cyanobacterial metabolites on *Chironomidae.* Cyanobacterial scums in natural environments consist of various strains simultaneously producing various secondary metabolites of different molecular structures.^34^ Different biotic or abiotic processes such as changes of hydraulic flashing or chemical treatment may facilitate decaying of cyanobacterial blooms.^35^ As a result, these compounds may be released in high amounts in a water environment and mixed with other compounds may affect the biota. These mixtures may manifest various total effects and the components may manifest as antagonistic, additive, or synergistic interactions, significantly differing from results obtained in experiments where animals are exposed solely to individual molecules. Consequently, our study aims to elucidate the effects of single AER-B, ANA-B, CYL, and their binary and ternary mixtures on the behavioral responses of *Chironomus* larvae, specifically focusing on immobilization, horizontal and vertical movement speed, and responses to light stimuli.

## 2 Materials and Methods

### 2.1 Experimental Animals

The test organism was *Chironomus aprilinus* at larval IVth instar stage purchased from a commercial supplier of biological materials, Kartinex (Sosnowiec, Poland). The larvae were acclimatized before the experiment by keeping them in artificial freshwater medium (Text S1) in the dark and at 20 °C for 24 h at a volume of 1 L with pH ranging between 7.4 ± 4 and conductivity between 330 ± 21 μS. The larvae were not fed before the experiment.

### 2.2 Chemicals and Experimental Design

We used pure cyanobacterial secondary metabolites: aeruginosin 98B (AER-B), anabaenopeptin-B (ANA-B), and cylindrospermopsin (CYL) (each >95% pure) purchased from a commercial biopharma supplier Enzo Life Sciences (Farmingdale). The stock solutions of the tested metabolites were prepared by adding 500 μL of methanol (MeOH, Avantor Poland, analytical grade) to the vial except for CYL, which was diluted in 500 μL of distilled water. The medium was prepared and used as a diluent for the preparation of working concentrations of the metabolites. The same amount of MeOH was used for preparation of vehicle control.

We used four nominal concentrations of each single metabolite. The three lowest concentrations of single metabolites (250, 500, and 1250 μg L^–1^) represented their environmental levels during cyanobacterial blooms. However, to calculate the 50% inhibitory concentration (IC_50_), we also exposed the larvae to higher levels (2500 μg L^–1^) of these compounds than those detected in the environment. Binary mixtures were used at four concentrations (1:1 ratio) and the sum concentrations of the two components were equal to the concentration of single metabolites. The sum of each of the four concentrations of the ternary mixture of three components was equal to the concentrations of single metabolites. Such an approach allowed to evaluate possible interactions of both components in mixtures.

#### 2.2.1 Concentrations of the Metabolites

*Chironomus* larvae were exposed to

(a) single cyanobacterial metabolites: AER-B, ANA-B, and CYL. Each compound was used at nominal concentrations:

- 250 μg L^–1^ (molar concentrations [m.c.]: 381.7 nM AER-B, 298.6 nM ANA-B, and 601.8 nM CYL)
- 500 μg L^–1^ (m.c.: 763.4 nM AER-B, 597.2 nM ANA-B, and 1203 nM CYL)
- 1250 μg L^–1^ (m.c.: 1908.5 nM AER-B, 1493 nM ANA-B, and 3009 nM CYL)
- 2500 μg L^–1^ (m.c.: 3817 nM AER-B, 2986 nM ANA-B, and 6018 nM CYL)

(b) their binary combinations: AER-B+ANA-B, AER-B+CYL, and ANA-B+CYL. The metabolites in each binary mixture were at the following concentrations:

- 125 μg L^–1^ + 125 μg L^–1^
- 250 μg L^–1^ + 250 μg L^–1^
- 625 μg L^–1^ + 625 μg L^–1^
- 1250 μg L^–1^ + 1250 μg L^–1^

(c) ternary combination of AER-B+ANA-B+CYL at three concentration variants, respectively:

- 83.3 μg L^–1^ + 83.3 μg L^–1^ + 83.3 μg L^–1^
- 166.6 μg L^–1^ + 166.6 μg L^–1^ + 166.6 μg L^–1^
- 416.6 μg L^–1^ + 416.6 μg L^–1^ + 416.6 μg L^–1^
- 833.3 μg L^–1^ + 833.3 μg L^–1^ + 833.3 μg L^–1^

The single cyanobacterial products and their binary or ternary mixtures were added to plastic square wells at appropriate amounts to form the final nominal concentrations at a volume of 4 mL. Each experimental and the control group consisted of 6 wells containing 2 larvae. To exclude the effects induced by MeOH itself, this solvent was also added to the vehicle control containers at the same concentrations as it was present at the highest concentration of the oligopeptides. However, the amount of MeOH did not exceed 0.01% of the total content of the cyanobacterial metabolite tested solution. The experimental animals were exposed for 72 h at 20° ± 1 C in darkness to eliminate the effect of light on the decomposition of cyanobacterial metabolites. The larvae were not fed during the short-time exposure to the metabolites.

### 2.3 Determination of Behavioral Parameters: Immobilization, Horizontal and Vertical Movement Speed, and Response to Light

#### 2.3.1 Immobilization

The immobilization assay was based on the OECD Acute Immobilization Test for *Chironomus*.^36^ The larvae placed in experimental plastic transparent containers were examined for immobilization at 72 h. Animals that did not show movement for 15 s after gentle stimulation with a thin wooden stick were treated as immobilized.

#### 2.3.2 Horizontal Movement Speed

After 15 min acclimation to light (4000 lx), vertical movement of larvae placed in each plastic square well was recorded for at least 1 min after 72 h exposure with a camera (Nikon D3100) mounted on a stand at a distance of 35 cm over the experimental wells. The recorded video clips were digitally analyzed by a frame-by-frame method with Tracker 4.11.0 software.^37^ Briefly, the horizontal displacement of the larvae was measured by selection of their position in separate video frames. After selection of the current position, the software automatically tracked the object to the last frame of the video clip. The measured movement speed of *Chironomus* larvae during 1 min recording was represented by amplitudograms plotted by the software. The mean of 6 individuals for the measured behavioral parameter was treated as a result for each experimental group.

#### 2.3.3 Vertical Movement

**Figure 1:**
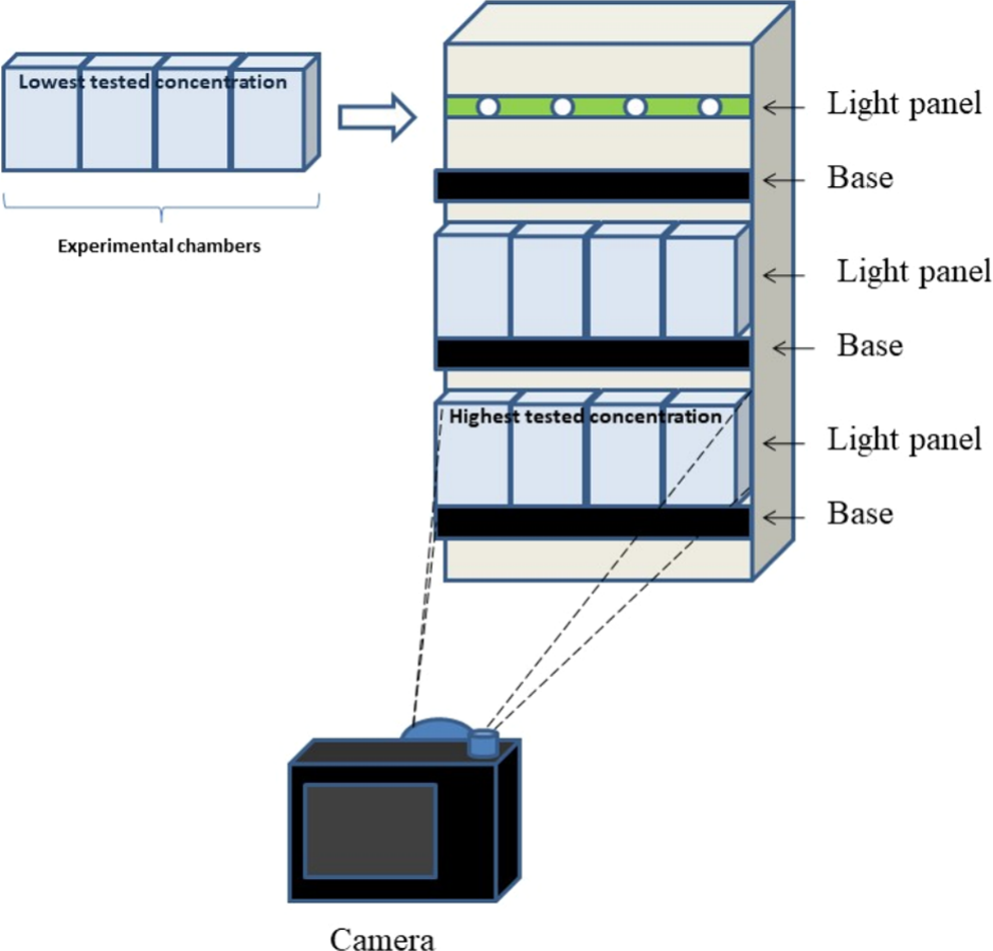
Apparatus constructed for video recording of *Chironomus* behavioral parameters: vertical movement and reaction to light.

For the determination of vertical swimming, 2 larvae for each experimental container were transferred to single transparent 6 mL plastic chambers containing 4 mL of the experimental medium placed in an apparatus equipped with a panel of LED white light source (4000 lx of light maximum intensity) (Figure 1). After 15 min acclimation, the animal movement was recorded with a video camera (Nikon D3100) mounted on a stand at a distance of 25 cm in front of the set of the experimental chambers. The response to light was determined by video recording of movement speed in the same chambers for 1 min before and 1 min after switching on the light panel in the experimental apparatus. The video clips were digitally analyzed by a frame-by-frame method with Tracker 4.11.0 software in the same manner as for horizontal movement. Vertical displacement of the larvae was measured (in millimeters per second) by selection of their position in separate video frames. The mean of 6 individuals for the measured behavioral parameter was treated as a result for each experimental group.

### 2.4 Calculation of Inhibitory Concentration, Combination Index, and Determination of the Interaction Type between Cyanobacterial Products

Inhibitory concentration (IC) for the behavioral parameters was calculated with the use of probit regression analysis. Maximum log likelihood estimation (null model −2 Log likelihood, full model −2 Log likelihood) was used as an overall fit model.

The types of interactions between the metabolites in binary mixtures (antagonistic, synergistic, or additive effects) were calculated by isobole analysis with the use of Compusyn (ver. 1.0) software. The interactions in the mixtures AER-B+ANA-B, ANA-B+CYL, and AER-B+CYL were determined with combination index (CI), which was calculated according to the following equations^38,39^^38,39^
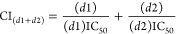
1
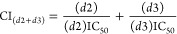
2
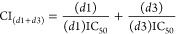
3Similarly, the following equation was used for a ternary mixture
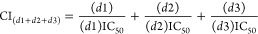
4where *d*_1_, *d*_2_, and *d*_3_ are the concentrations of AER-B, ANA-B, and CYL, respectively, in a binary mixture inducing 50% effect (IC_50_): IC_50_ is the half-maximum effective concentrations of the single AER-B, ANA-B, and CYL, respectively. Loewe additivity model was used^40^ for the calculation of possible additive effects according to the following equation
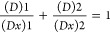
5where (*D*)1 and (*D*)2 are the combination concentrations of metabolite 1 and metabolite 2 inducing a 50% inhibitory effect, whereas (*Dx*)1 and (*Dx*)2 correspond to the concentrations of the single metabolite 1 and metabolite 2 with the same IC_50_. The interaction between two metabolites in binary mixtures was expressed graphically. The IC_50_ values for each of the single oligopeptides being a component of a binary mixture were marked respectively on the *x* and *y* axes. The plotted line (additivity line) connecting the two points of the IC_50_ values is the representation of the additive interaction of the two chemicals. The interaction is considered as synergistic when the combination index (CI) < 1, additive effect when CI = 1, and antagonistic effect at CI > 1. The interaction was calculated by Compusyn (ver. 1) software according to the following equation by Chou and Talalay^41^^41^
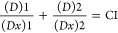
6Polygonograms plotted by the software presented the CI values between the metabolites in the ternary mixtures. The level of interaction based on the range of the calculated CI was represented by symbols according to Chou.^42^

### 2.5 Statistical Analysis

Statistical analyses were done with the use of Statistica 13.1 software. Homogeneity and normality of variances were determined with Levene’s and Shapiro–Wilk’s tests, respectively. The comparisons of mean values among the groups were done by one-way ANOVA (significant differences between the concentrations of the tested cyanobacterial products) followed by Dunnett’s post hoc test to compare data in the experimental and the control groups. The data were treated as significant at *p* < 0.05. The results are shown as means ± standard deviation (SD). Pearson correlation coefficients (*r*) were used to evaluate the relationships between the horizontal and vertical movement speed based on IC_50_ for the two parameters, at *p* < 0.05.

## 3 Results

### 3.1 Immobilization

**Figure 2:**
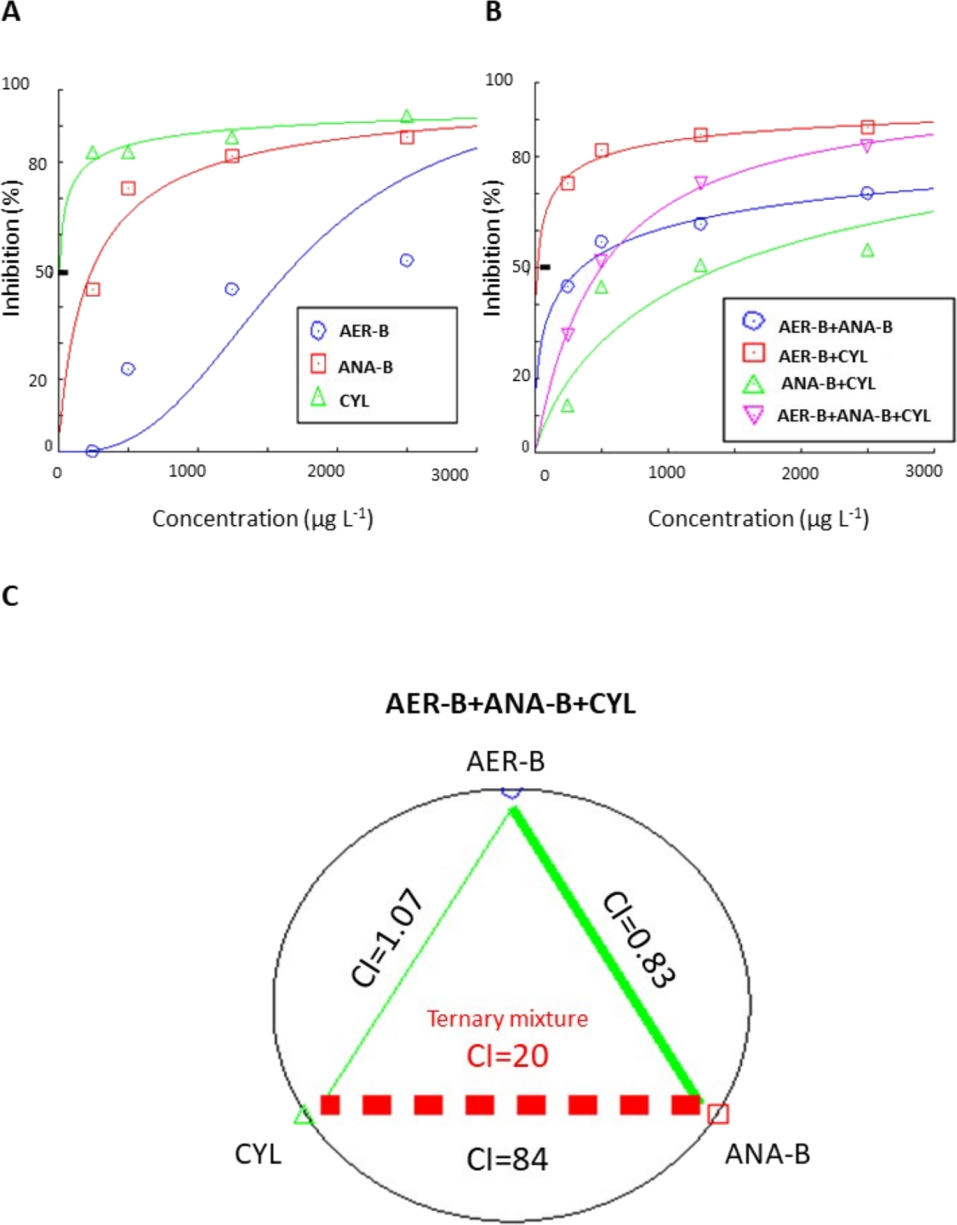
(A) Concentration–response relationship for immobilization of *C. aprilinus* larvae exposed to single cyanobacterial metabolites aeruginosin-B (AER-B), anabaenopeptin-B (ANA-B), and cylindrospermopsin (CYL). (B) Concentration–response relationship for immobilization of the larvae exposed to binary and ternary mixtures of the cyanobacterial metabolites. (C) Type of interactions of the binary and ternary combinations of the metabolites on larvae immobilization. Thin and thick green lines represent nearly additive and slight synergistic effects, respectively. The red dashed line with CI values represents antagonistic interactions; *n* = 6.

**Figure 3:**
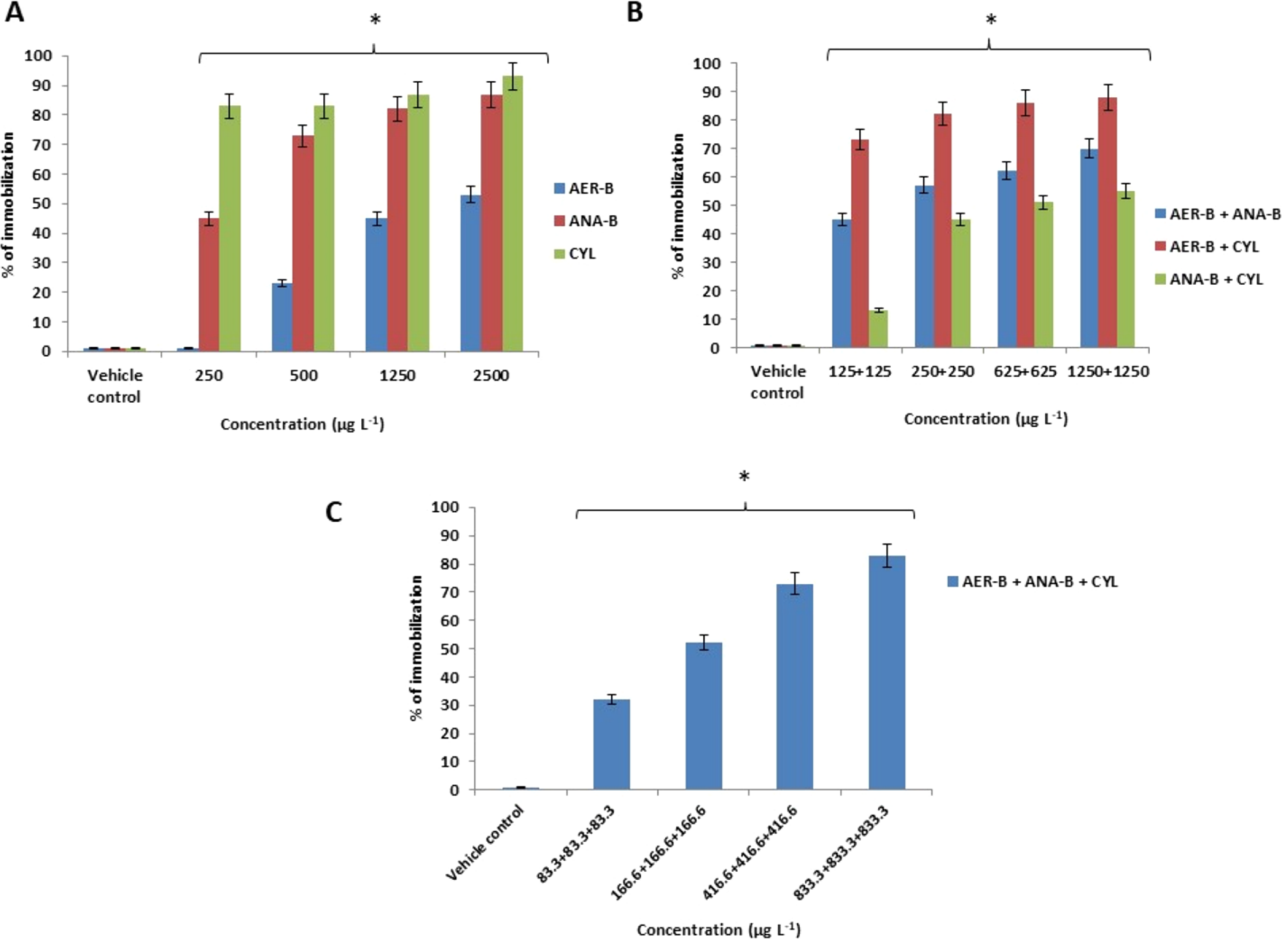
(A) Percentage of the immobilized *Chironomus* larvae after exposure to single cyanobacterial metabolites aeruginosin-B (AER-B), anabaenopeptin-B (ANA-B), and cylindrospermopsin (CYL). (B) A percentage of the immobilized larvae after exposure to their binary mixtures of the metabolites. (C) A percentage of the immobilized larvae after exposure to the ternary combination of the metabolites; *n* = 6. Asterisks indicate statistical significance at *p* < 0.05.

The study showed that 93 ± 7% of the immobilized *Chironomus* larvae were found at 2500 μg L^–1^ of CYL (Figures 2A and 3A). However, a lower percentage of nonmotile animals (53 ± 5%) was found in the group challenged with AER-B. 88 ± 5% of the larvae were immobilized in the binary mixture 1250 μg L^–1^ AER-B+1250 μg L^–1^ CYL (Figures 2B and 3B). 83 ± 6% of the immobilized animals were noted at the highest concentration of the ternary mixture of the metabolites with very strong antagonistic effects (CI = 20) (Figure 2C).

### 3.2 Horizontal Movement Speed

**Figure 4:**
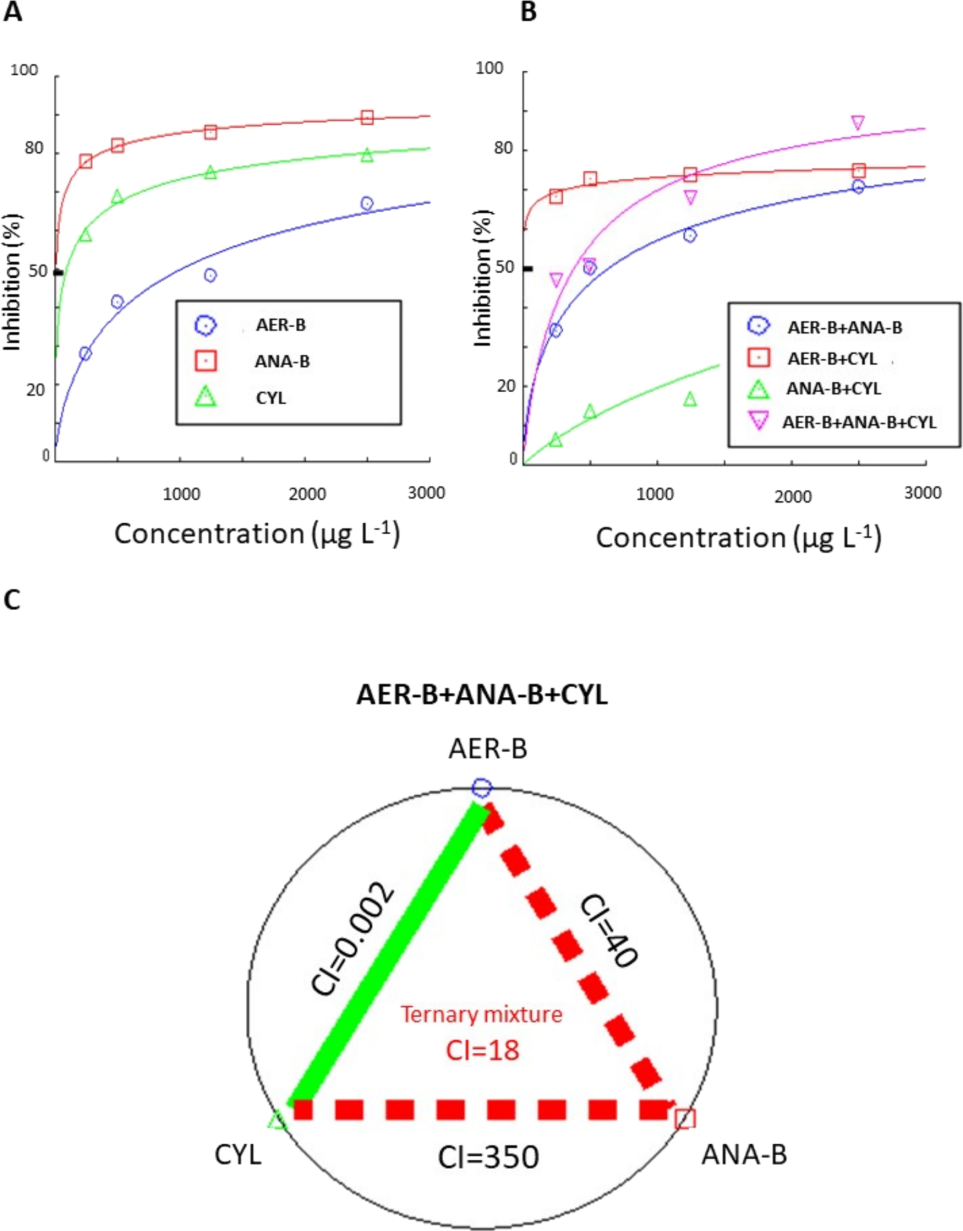
(A) Concentration–response relationship of single cyanobacterial metabolites aeruginosin-B (AER-B), anabaenopeptin-B (ANA-B), and cylindrospermopsin (CYL) on horizontal movement speed of *C. aprilinus* larvae. (B) Concentration–response relationship of the binary combinations of the metabolites on horizontal movement speed. (C) Type of interactions of the binary and ternary combinations of the metabolites on horizontal movement speed. Red dashed and green lines with CI values represent antagonistic and very strong synergistic interactions, respectively; *n* = 6.

**Figure 5:**
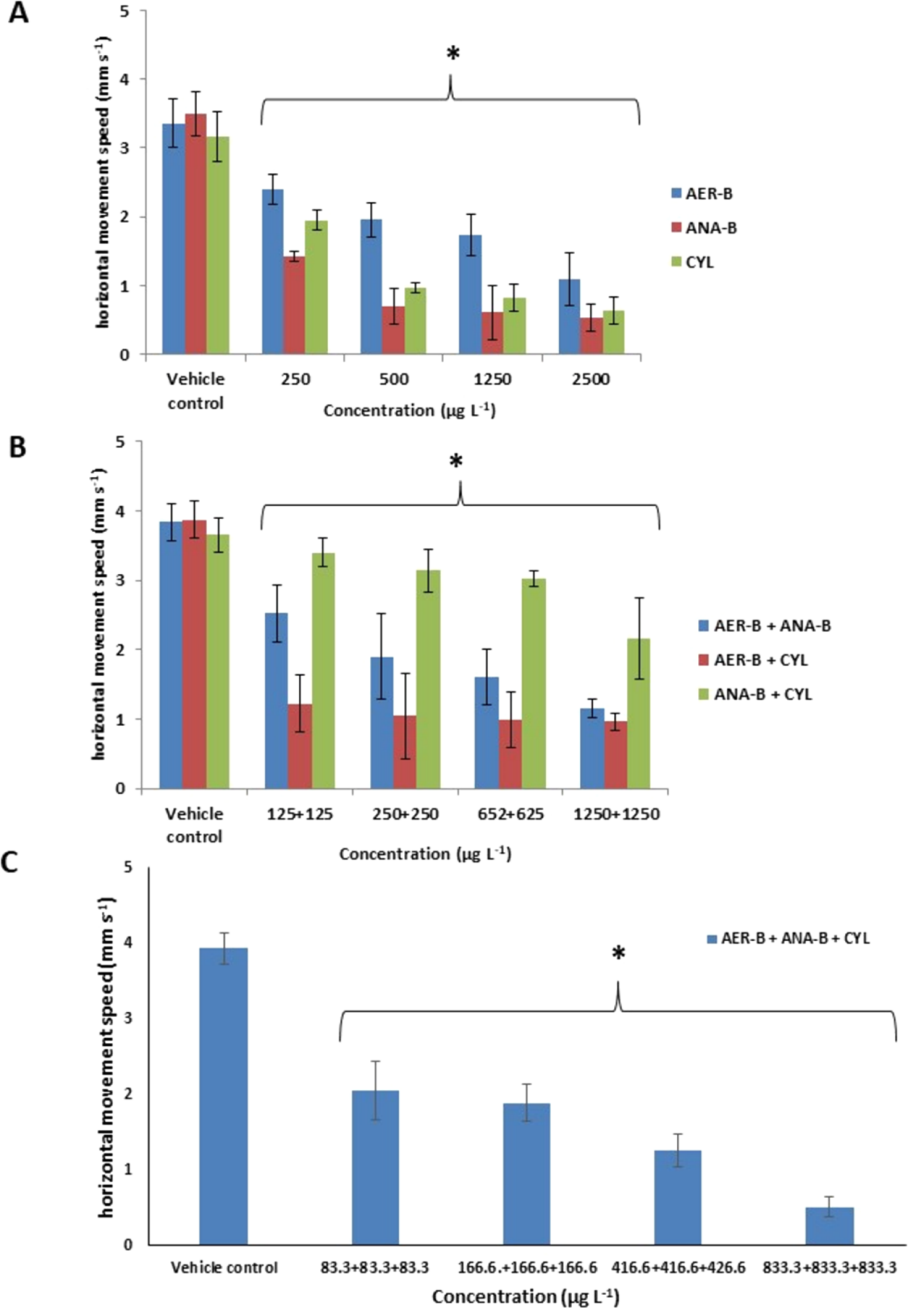
(A) Horizontal movement speed of *C. aprilinus* larvae exposed to single cyanobacterial metabolites aeruginosin-B (AER-B), anabaenopeptin-B (ANA-B), and cylindrospermopsin (CYL). (B) Horizontal movement speed of the larvae exposed to the binary combinations of the metabolites. (C) Horizontal movement speed of the larvae exposed to the ternary mixtures of the metabolites; *n* = 6. Asterisks indicate statistical significance at *p* < 0.05.

The test revealed that horizontal movement speed was decreased by each tested single cyanobacterial metabolite (Figures 4A and 5A). ANA-B was calculated to be the most potent inhibitor with IC_50_ = 8.0 ± 0.8 μg L^–1^ (Table S1). This parameter was 0.54 ± 0.2 mm s^–1^ in the group exposed to 2500 μg L^–1^ of this oligopeptide when compared with the control (3.5 ± 0.32 mm s^–1^). The binary mixtures also reduced this movement parameter (Figure 5B). The mixture AER-B+CYL showed the highest inhibitory potential (IC_50_ = 0.43 ± 0.003 μg L^–1^) (Figure 4B and Table S1) with a very strong synergistic interaction (CI = 0.002) (Figure 4C). The highest inhibition was found in the group exposed to 1250 μg L^–1^ AER-B+1250 μg L^–1^ CYL (0.96 ± 0.13 mm s^–1^) when compared with the control (3.87 ± 0.27 mm s^–1^). On the other hand, the components in the mixture of ANA-B+CYL manifested a very strong antagonistic interaction with CI = 350 (Figure 4C). The ternary mixture showed the strongest reduction of horizontal movement speed at its highest concentration (0.51 ± 0.13 mm s^–1^) when compared with the single metabolites, binary mixtures, and the control (3.93 ± 0.21 mm s^–1^) (Figure 5C); however, the IC_50_ (375 ± 56 μg L^–1^) was higher than that of AER+CYL (Table S1). The value of CI = 18 in the ternary mixture suggests that the components showed very strong antagonistic interactions (Figure 4C).

### 3.3 Vertical Movement Speed

**Figure 6:**
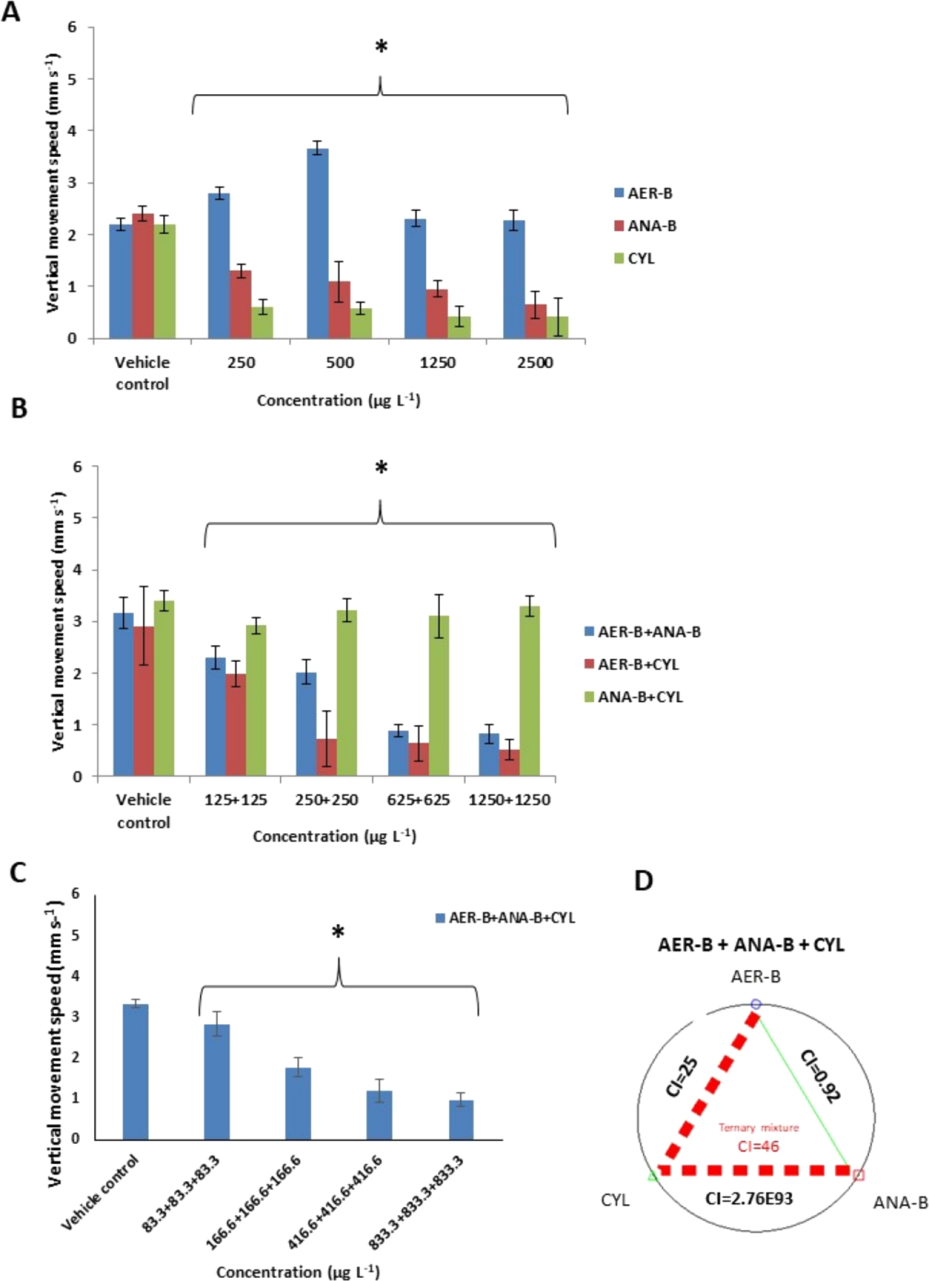
(A) Vertical movement speed of *C. aprilinus* larvae exposed to single cyanobacterial metabolites aeruginosin-B (AER-B), anabaenopeptin-B (ANA-B), and cylindrospermopsin (CYL). (B) Vertical movement speed of the larvae exposed to the binary combinations of the metabolites. (C) Vertical movement speed of the larvae exposed to the ternary mixtures of the metabolites; *n* = 6. Asterisks indicate statistical significance at *p* < 0.05. (D) Green and red dashed lines with CI values represent nearly additive and very strong antagonistic interactions, respectively.

The study showed various effects of single cyanobacterial metabolites on the vertical movement speed of *C. aprilinus* larvae (Figure 6A). Although this parameter was increased in the invertebrates exposed to AER-B at 250 and 500 μg L^–1^ (2.8 ± 0.12 and 3.67 ± 0.13 mm s^–1^, respectively), when compared with the control (2.2 ± 0.13 mm s^–1^), the larvae challenged with ANA-B and CYL showed the reduced speed with the lowest value at 2500 μg L^–1^ (0.65 ± 0.26 and 0.42 ± 0.36 mm s^–1^, respectively) when compared with the respective control groups (2.4 ± 0.14 and 2.2 ± 0.18 mm s^–1^). IC_50_ values indicated that CYL had the highest inhibitory potential (6.41 ± 2 μg L^–1^) (Table S1). The binary mixtures 1250 μg L^–1^ AER-B+1250 μg L^–1^ ANA-B and 1250 μg L^–1^ AER+1250 μg L^–1^ CYL strongly reduced the speed of vertical movement (0.82 ± 0.21 and 0.51 ± 0.22 mm s^–1^) when compared with the respective control groups (3.17 ± 0.3 and 2.91 ± 0.76 mm s^–1^); however, ANA-B+CYL had no effect (Figure 6B). The ternary mixture of the tested cyanobacterial metabolites at the highest concentration (833.3 μg L^–1^ AER-B+833.3 μg L^–1^ ANA-B+833.3 μg L^–1^ CYL) induced a significant inhibition of the vertical movement speed (0.98 ± 0.16 mm s^–1^) in a concentration-dependent manner (*p* < 0.01) when compared with the control (3.34 ± 0.1 mm s^–1^) (Figure 6C). The mixture AER-B+ANA-B induced nearly additive effects (CI = 0.92); however, the two other binary mixtures and the ternary one showed very strong antagonistic interactions (Figure 6D).

### 3.4 Correlation between Horizontal and Vertical Movement Speed

Although most of the single metabolites and their binary and ternary combinations had a strong positive correlation between horizontal and vertical movement speed (*r* = 0.77–0.99; *p* < 0.01), a slight negative correlation was found regarding the two parameters of larvae exposed to single AER-B (*r* = −0.45; *p* < 0.01) (Table S2).

### 3.5 Response to Light

**Figure 7:**
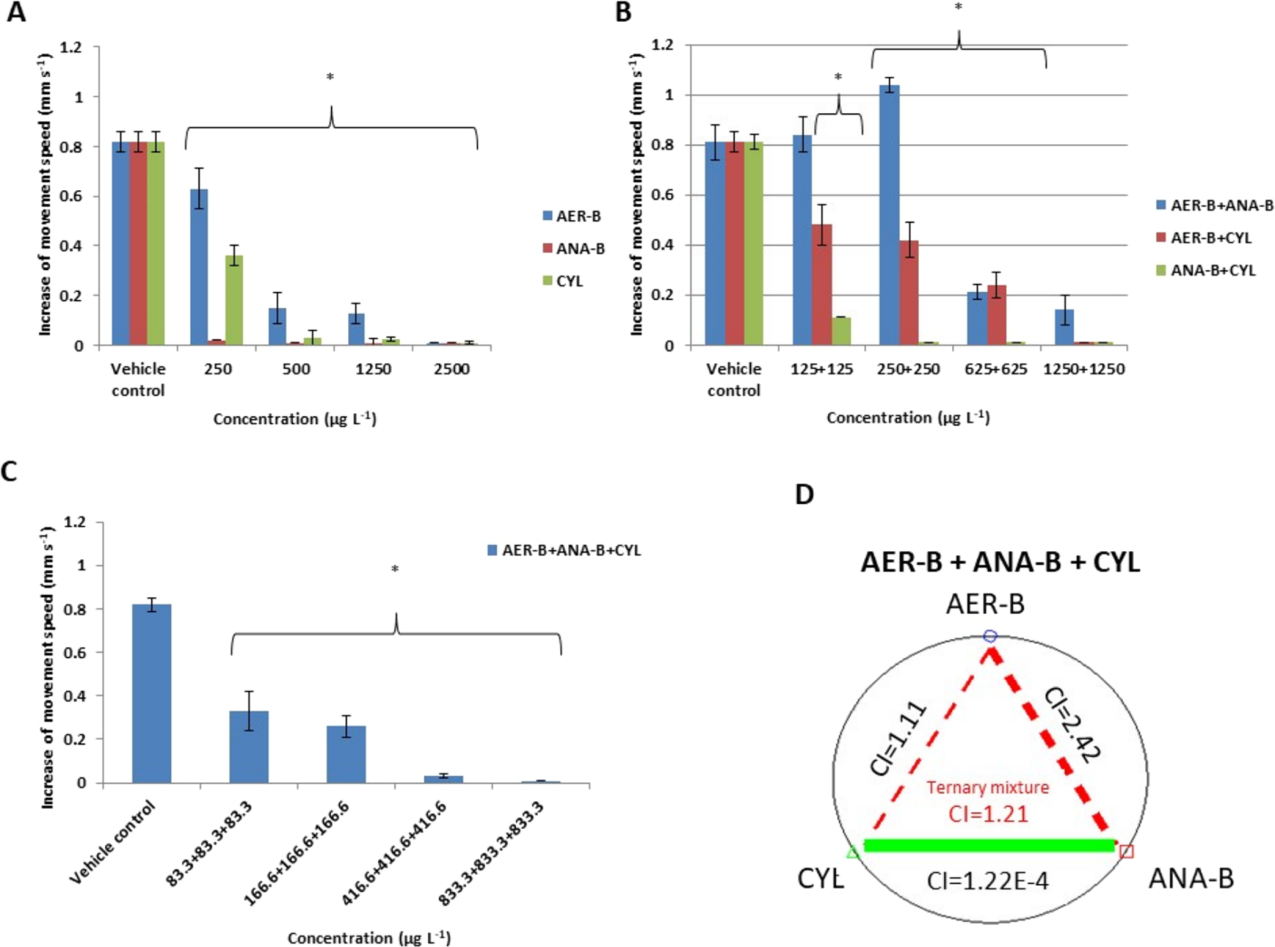
(A) Response to light of *C. aprilinus* larvae exposed to single cyanobacterial metabolites aeruginosin-B (AER-B), anabaenopeptin-B (ANA-B), and cylindrospermopsin (CYL). (B) Response to light of the larvae exposed to the binary combinations of the metabolites. (C) Response to light of the larvae exposed to the ternary mixtures of the metabolites; *n* = 6. Asterisks indicate statistical significance at *p* < 0.05. (D) Green and red dashed lines with CI values represent very strong synergistic and antagonistic effects, respectively.

We found that the control larvae increased their motility after light stimulation in the apparatus by 0.82 ± 0.04 mm s^–1^ (Figure 7A). On the other hand, response to light was inhibited in larvae exposed to each concentration of ANA-B and CYL. The two highest concentrations of these metabolites completely ceased the response to light. Although the reaction to light in the binary mixture of AER-B+ANA-B at 125 + 125 μg L^–1^ was similar to that in the control group, it was stimulated at 250 + 250 μg L^–1^ (increase by 1.04 ± 0.03 mm s^–1^) (Figure 7B). The lower level of responsiveness to light when compared with the control group was observed in larvae exposed to the mixtures AER+CYL at a concentration of 125 + 125 μg L^–1^ (increase of movement as low as by 0.48 ± 0.08 mm s^–1^); however, those at all of the tested concentrations of ANA-A+CYL and the highest concentration of AER+CYL did not show changes in the measured parameter when compared with the control. The ternary mixture completely ceased the reaction to light of the experimental larvae but only at the highest concentrations of the components (833.3 + 833.3 + 833.3 μg L^–1^) (Figure 7C) with moderately antagonistic interactions (CI = 1.21) (Figure 7D and Table S1).

## 4 Discussion

The results of our study demonstrate that the movement behavior of *C. aprilinus* larvae is significantly affected by the exposure to individual cyanobacterial metabolites (AER-B, ANA-B, and CYL) as well as their binary or ternary combinations. Notably, ANA-B and CYL, when administered individually, induced a pronounced immobilization, which possibly may contribute to their interactions with neuromodulatory peptides or receptors responsible for larval locomotor activity. CYL seems to be the most potent immobilizing agent, possibly by the fact that this alkaloid is easily distributed in the larvae and it may also interact with many biochemical targets^25^ attributable to its neurotoxic activity.^43−45^ However, when these metabolites were combined in a binary mixture, a remarkable decrease in their potential to immobilize individuals was observed. This reduction was particularly evident in the case of the ANA-B and CYL combination and the ternary mixture, which exhibited a high level of antagonism. Strikingly, the AER-B+CYL combination evoked a nearly additive effect, and AER-B+ANA-B a slight synergistic interaction indicating a complex interplay of these metabolites on the movement behavior of the larvae. Different levels of immobilization observed at the same concentrations of the tested metabolites in the mixtures may result from their various biochemical interactions with structures responsible for the locomotory functions of *Chironomus* larvae. Furthermore, ANA-B acted as an antagonizer to CYL when it was a component of the binary mixture, mitigating to some extent immobilizing effects of the alkaloid. This intricate relationship between CYL and some oligopeptides underscores the importance of considering interactive effects in assessing the impact of cyanobacterial metabolites on *C. aprilinus* larvae using immobilization as a biomarker of toxicity.

While the effects of cyanobacterial metabolites on *Chironomus* motility have been less explored, our results find resonance in studies on other aquatic organisms. For instance, *Daphnia magna* exposed to the neurotoxic cyanobacterial alkaloid anatoxin-a exhibited disturbances in swimming behavior.^37^ The effects were observed at much higher concentrations of the cyanobacterial alkaloid (at a range of 5–50 mg L^–1^) than those of the two oligopeptides and CYL in our experiments. Similarly, in another study, cyclic oligopeptides, including ANA-B and microcystin-LR (MC-LR,) were found to immobilize crustaceans such as *D. magna*(16) at concentrations 0.5–5 mg L^–1^, which are similar to those used in the present investigation. Additionally, the impact of anabaenopeptins on diverse organisms, including the nematode *Caenorhabditis elegans*(15) at 10 μg L^–1^, the rotifer *Brachionus calyciflorus*(9) (a range of 100–2500 μg L^–1^), and the amoeba *Acanthamoeba castellanii*(46) (a concentration range of 0.1–1000 μg L^–1^), further emphasizes the wide-ranging effects of cyanobacterial metabolites on various aquatic species. The abovementioned studies also indicate that different levels of sensitivity to various cyanobacterial metabolites may be found in aquatic invertebrates.

The findings of our study also unveil a notable reduction in the horizontal movement speed of *Chironomus* larvae when exposed to individual cyanobacterial products. The inhibition of this parameter may be a result of the interaction of the tested metabolites with protein neuromodulators involved in locomotory activity or blocking of specific receptors in the neuronal synapses and/or neuromuscular junctions. Particularly CYL and ANA-B exhibited stronger inhibitory effects on motility when administered individually compared with their binary combinations, where antagonistic effects were evident. Antagonistic effects induced by this binary mixture may be explained by the fact that these two metabolites may form complexes decreasing the ability of each other to affect larval horizontal locomotion. The elucidation of the underlying mechanisms responsible for this phenomenon warrants further investigation, as it remains a complex and intriguing aspect of cyanobacterial metabolite impact on *Chironomus* larvae.

Several authors have previously employed *Chironomus* larvae movement as an indicator of toxicity,^47−50^ often assessing the larvae’s ability to perform three normal figure-eight swimming motions when manipulated with forceps. Our study builds on this foundation, emphasizing the utility of larval movement as a sensitive indicator of toxicity induced by cyanobacterial metabolites.

In addition to horizontal movement speed, our study explored the impact of cyanobacterial metabolites on vertical motility. Most single cyanobacterial metabolites (except for AER-B) inhibited this end point, when compared with the control group, showing a strong positive correlation with horizontal movement speed. This finding suggests that natural exposure of *Chironomus* larvae to some cyanobacterial metabolites may result in disturbances of multidirectional movement specific to this biological model. However, intriguingly, AER-B at lower concentrations stimulated vertical motility; therefore, it may be speculated that on the molecular level, single ANA-B may stimulate some parts of the locomotor system such as excitatory synapses specifically responsible for larval vertical motility. Moreover, it seems that nearly additive effects found in the mixture AER-B+ANA-B may be a result of the strong mitigation of AER-B toward the stimulatory activity of ANA-B. Furthermore, the response to light, an essential behavioral parameter, was altered in both the single metabolite-exposed larvae and those exposed to binary and ternary mixtures. Although it is possible that the single metabolites inhibited the activity or synthesis of the neuromodulatory peptides or proteins related to neuromuscular transmission involved in processes associated with larval reaction to light, the mixtures induced varied responses, from stimulation (AER-B+ANA-B) to a high level of inhibition with very strong synergistic effects (ANA-B+CYL). This suggests a possibility of a substantial variety of interactions between the components on the molecular level (inhibition activity of peptides, blocking or activating the receptors in neuromuscular junctions), which may affect the total toxicity of mixtures. Based on these results, we may assume that in natural conditions, cyanobacterial metabolites and their mixtures may alter the behavioral response to light in *Chironomus* larvae. As a consequence, increased susceptibility to predators or discrepancies in some larval functions such as burrowing behavior and feeding may occur. Although light responses have previously been utilized as an indicator of insect behavior,^27,28^ our experiments on *Chironomus* larvae suggest its applicability also in ecotoxicological testing. Moreover, the novel apparatus used in our study allowed for the simultaneous measurement of two critical parameters in *Chironomus* larvae: vertical movement and response to light. These parameters demonstrated high sensitivity to cyanobacterial metabolites, reinforcing their suitability for ecotoxicological testing in aquatic environments.^51^

The results of our study provided some data that may justify further investigations on the mechanisms of behavioral alterations. Results from the previous experiments on snail neurons and aquatic vertebrates, such as tilapia,^44^ suggest that CYL-induced behavioral changes in *Chironomus* larvae may be probably linked to neurotoxicity. The relatively small size of CYL molecule enables passive transport through cell membranes, potentially leading to quick distribution within the larval nervous system and inducing neurobehavioral alterations, possibly through interference with various neuromodulator machineries such as the acetylcholine system.^43^ On the other hand, the tested oligopeptides, known as inhibitors of some proteases in mammalian cells,^52^ may inhibit the production of some neuromodulators or directly interact with neuromuscular transmission and thus impair *Chironomus* larvae behavior, for example, by influencing or mimicking norepinephrine, a crucial neurotransmitter in neurobehavioral processes.^53^

Our laboratory experiments with the combination index approach revealed both antagonistic and synergistic effects for binary and ternary mixtures of cyanobacterial metabolites. It suggests that the total effects may be different and dependent on the structural type of metabolites and their concentration in mixtures. Although the exposure scenario in our study assumed equal concentrations of metabolites as components in each mixture, it may not be fully relevant to naturally occurring situations since the real amounts of these mixed compounds in the natural environment may be varied. It is known that synergism may be often found in mixtures of these compounds when they are released at higher amounts from the decaying cyanobacterial cells in mixtures reaching high concentrations in water;^54^^54^ however, lower levels of these compounds inducing additive interactions may be present more frequently. It is also equally possible that the animals in the aquatic environment are exposed to mixtures of chemicals possessing various mechanisms of action or cumulative effects. It suggests that the concentration addition model may be more suitable for the determination of mixture toxicity for such assumptions.

In conclusion, our study provides compelling evidence that the behavior of *C. aprilinus* larvae is significantly influenced by single cyanobacterial metabolites and their binary or ternary combinations. Also, the results suggest that the total effects of mixtures may depend on the concentration and the structural type of their components. This suggests potential hazards to benthic larvae of certain insect species, with subsequent ecological consequences. In a natural scenario, *Chironomus* larvae may respond to cyanobacterial metabolites especially during bloom senescence, with potential physiological and ecological repercussions such as increased vulnerability to predators or disturbances of burrowing behavior. The behavioral parameters of *Chironomidae* larvae emerge as valuable tools in ecotoxicological assessment, offering insights into the impacts of cyanobacterial metabolites on aquatic ecosystems.^55^

## Supporting Information Available

Artificial water composition (Text S1); inhibitory concentrations (IC_50_) (μg L^–1^ ± standard deviation) and combination index (CI) values of the tested parameters in *C. aprilinus* larvae exposed to cyanobacterial metabolites aeruginosin-B (AER-B), anabaenopeptin-B (ANA-B), and cylindrospermopsin (CYL), and their binary and ternary mixtures (Table S1); Pearson correlation coefficient (*r*) between horizontal and vertical movement speed of *C. aprilinus* larvae exposed to cyanobacterial metabolites aeruginosin-B (AER-B), anabaenopeptin-B (ANA-B), and cylindrospermopsin (CYL), and their binary and ternary mixtures; *p* < 0.01 (Table S2) (PDF)
