# Supplementary material for: Behavioral Responses of Chironomus aprilinus Larvae as Proxies for Cyanobacterial Metabolite Interactions: Insights from Ternary Combinations
Source: Environ Sci Technol. 2024 Oct 15;58(43):19199–210. doi: 10.1021/acs.est.4c07823 (PMC11526367; doi:10.1021/acs.est.4c07823)
Supplement: Supplementary file 1 — es4c07823_si_001.pdf [file es4c07823_si_001.pdf]

Supporting information for:

Behavioral responses of *Chironomus aprilinus* larvae as proxies for cyanobacterial metabolite interactions: insights from ternary combinations

Adam Bownik[1*], Donald Wlodkowic[2], Barbara Pawlik-Skowrońska[1], Tomasz Mieczan[1]

[1]Department of Hydrobiology and Protection of Ecosystems, University of Life Sciences in Lublin, Dobrzańskiego 37, 20-262, Lublin, Poland

[2]The Neurotox Lab, School of Science, RMIT University, Plenty Road, P.O. Box 71, Bundoora, VIC 3083, Australia

Submitted to :

Environmental Science & Technology

Number of pages (including this page) 4

Number of texts: 1

Number of tables: 2

Text S1. The artificial water composition:

64.75 mg L^{-1} $NaHCO_3$, 294 mg L^{-1} $CaCl_2.2H_2O$, 123.25 mg L^{-1} $MgSO_4.7H_2O$ 5.75 mg L^{-1} KCl

Table S1. Inhibitory concentrations (IC50) (µg L^{-1}±Standard Deviation) and Combination Index (CI) values of the tested parameters in *Chironomus aprilinus* larvae exposed to cyanobacterial metabolites aeruginosin-B (AER-B), anabaenopeptin-B (ANA-B) and cylindrospermopsin (CYL), their binary and ternary mixtures. 72 h-IC50 calculated using the probit-regression analysis are predictive values. N.C.–not calculable, VSA-very strong antagonism, A-antagonism, MA-moderate antagonism, NAD-nearly additive, SlS-slight synergism, MS-moderate synergism, S-synergism, SS-strong synergism, VSS-very strong synergism[42]

**parameter tested**	**single cyanobacterial metabolites**			**binary mixtures of metabolites**			**ternary mixture of metabolites**
	**AER-B**	**ANA-B**	**CYL**	**AER-B+ANA-B**	**AER-B+CYL**	**ANA-B+CYL**	**AER-B+ANA-B+CYL**
**immobilization**	1650±52	234±97	8.49±0.2	343±5 **CI=0.83 (SlS)**	18±1 **CI=1.07 (NAD)**	1390±67 **CI=84.8 (VSA)**	493±35 **CI=20 (VSA)**
**horizontal movement speed**	1000±17	8±0.8	90±15	627±69 **CI= 40.5 (VSA)**	0.43±32 **CI=0.002 (VSS)**	5032±156 **CI=350 (VSA)**	375±56 **CI= 18 (VSA)**

**vertical movement speed**	N.C.	390 ±5	6.41± 2	719±45 **CI=0.92 (NAD)**	324±14 **CI=25 (VSA)**	N.C. **CI=2.76E 93 (VSA)**	877±154 **CI=46 (VSA)**
**response to light**	398±23	240 ±12	342±2 4	727±23 **CI=2.42 (A)**	409±37 **CI=1.11 (MA)**	0.03±0.01 **CI=1.22E -4 (VSS)**	379±29 **CI=1.21 (MA)**

Table S2. Pearson correlation coefficient (r) between horizontal and vertical movement speed of *Chironomus aprilinus* larvae exposed to cyanobacterial metabolites aeruginosin-B (AER-B), anabaenopeptin-B (ANA-B) and cylindrospermopsin (CYL), their binary and ternary mixtures; $p<0.01$.

**Parameter tested**	**Single cyanobacterial metabolites**			**Binary mixtures of metabolites**			**Ternary mixture of metabolites**
	**AER-B**	**ANA-B**	**CYL**	**AER-B+ANA-B**	**AER-B+CYL**	**ANA-B+CYL**	**AER-B+ANA-B+CYL**
**Horizontal vs vertical**	-0.46	0.86	0.77	0.91	0.98	0.72	0.86

**movement speed**							
